# Nebulin: big protein with big responsibilities

**DOI:** 10.1007/s10974-019-09565-3

**Published:** 2020-01-25

**Authors:** Michaela Yuen, Coen A. C. Ottenheijm

**Affiliations:** 1 Department of Physiology, Amsterdam UMC, Location VU Medical Center, Amsterdam, The Netherlands; 2 Discipline of Paediatrics and Child Health, Faculty of Medicine, University of Sydney, Sydney, Australia

**Keywords:** Nebulin, Thin filament, Nemaline myopathy, Cross-bridge cycling, Actin

## Abstract

Nebulin, encoded by *NEB*, is a giant skeletal muscle protein of about 6669 amino acids which forms an integral part of the sarcomeric thin filament. In recent years, the nebula around this protein has been largely lifted resulting in the discovery that nebulin is critical for a number of tasks in skeletal muscle. In this review, we firstly discussed nebulin’s role as a structural component of the thin filament and the Z-disk, regulating the length and the mechanical properties of the thin filament as well as providing stability to myofibrils by interacting with structural proteins within the Z-disk. Secondly, we reviewed nebulin’s involvement in the regulation of muscle contraction, cross-bridge cycling kinetics, Ca^2+^-homeostasis and excitation contraction (EC) coupling. While its role in Ca^2+^-homeostasis and EC coupling is still poorly understood, a large number of studies have helped to improve our knowledge on how nebulin affects skeletal muscle contractile mechanics. These studies suggest that nebulin affects the number of force generating actin-myosin cross-bridges and may also affect the force that each cross-bridge produces. It may exert this effect by interacting directly with actin and myosin and/or indirectly by potentially changing the localisation and function of the regulatory complex (troponin and tropomyosin). Besides unravelling the biology of nebulin, these studies are particularly helpful in understanding the patho-mechanism of myopathies caused by *NEB* mutations, providing knowledge which constitutes the critical first step towards the development of therapeutic interventions. Currently, effective treatments are not available, although a number of therapeutic strategies are being investigated.

## Nebulin in muscle disease

**Fig. 1:**
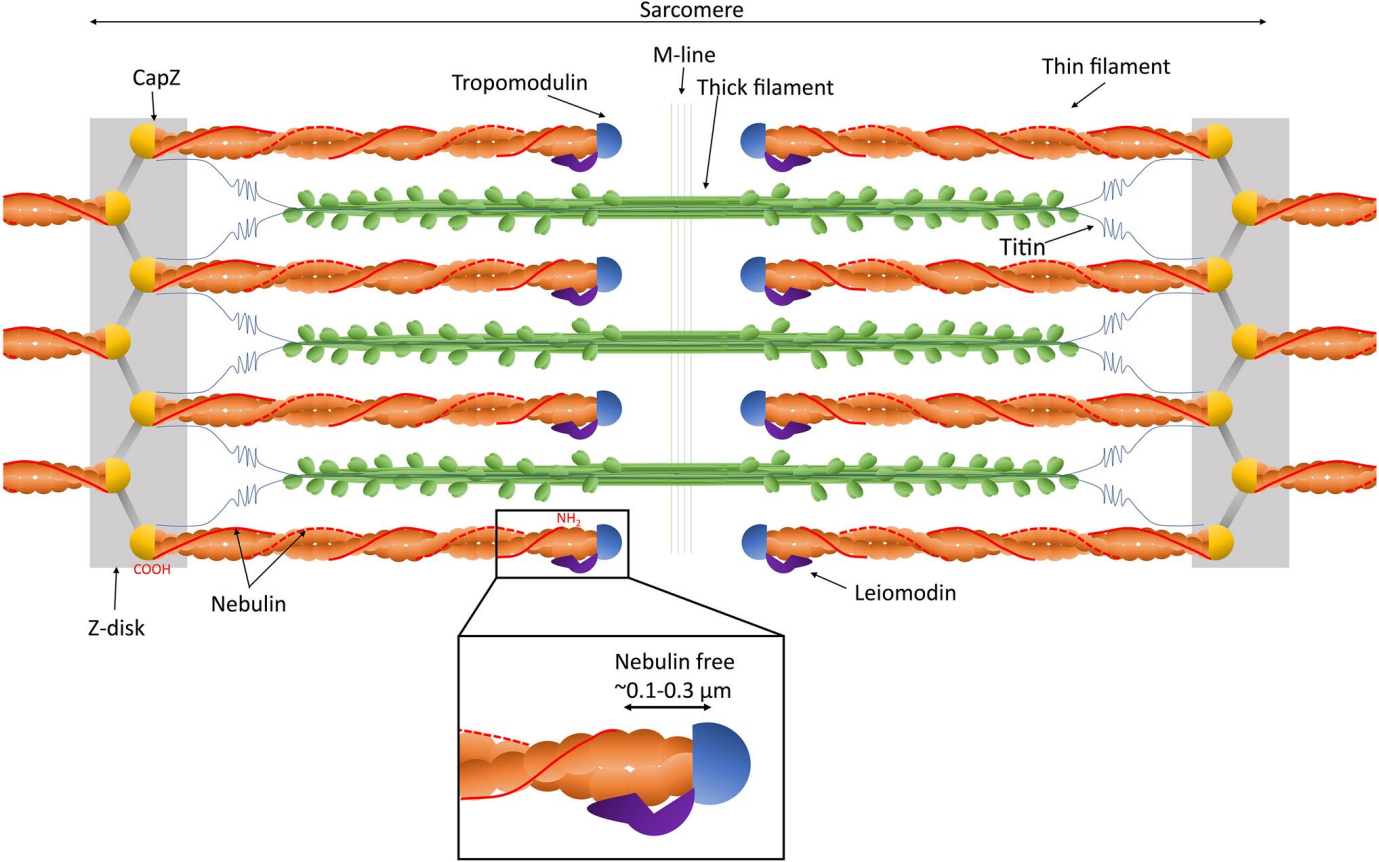
Nebulin in the skeletal muscle sarcomere. The structure and major components of the skeletal muscle sarcomere are illustrated. For the purpose of this illustration, it was assumed that two nebulin molecules (shown in red) are incorporated in each skeletal muscle thin filament. Nebulin’s C-terminus is anchored in the Z-disk (from M182; Millevoi et al. 1998) and interacts with the thin filament capping protein CapZ. Nebulin then extends alongside the thin filament towards the center of the sarcomere. The N-terminus of nebulin localises close to the thin filament pointed end, leaving the last ~ 0.1–0.3 µm of the thin filament nebulin free (see inset). (Color figure online)

Nebulin, encoded by *NEB*, is a giant actin filament associated protein (Fig. 1). It was first discovered in 1980 (Wang and Williamson 1980) and described as an inextensible filament working in parallel with titin in skeletal muscle (Wang and Wright 1988). Its critical role in muscle function became apparent when mutations in *NEB* were associated with autosomal recessive nemaline myopathy, a disease characterised by generalised skeletal muscle weakness and the presence of electron dense protein accumulations (nemaline rods) on patient muscle biopsy examination (after *TPM3*, *NEB* was the second gene associated with this condition) (Pelin et al. 1999).

The clinical severity of pathogenic mutations in *NEB* was found to vary from severe, neonatally lethal muscle weakness to mild disease with childhood onset (Pelin et al. 1999, 2002; Wallgren-Pettersson et al. 2002, 2004). The disease severity was suggested to correlate with the amount of nebulin protein expressed in patient muscle—less protein was associated with a more severe phenotype (de Winter et al. 2013; Lawlor et al. 2011; Ochala et al. 2011; Ottenheijm et al. 2009, 2010).

Although it was soon recognised that *NEB* mutations are a major cause of nemaline myopathy (accounting for ~ 50% of cases, Romero et al. 2013), its large size (183 exons; Donner et al. 2004) made sequencing and sequence analysis challenging. Since protein levels are reduced in many patients (e.g. Ottenheijm et al. 2009), or mutations result in expression of a truncated protein, western blot analysis was useful to direct genetic testing, although it was not always straight forward (Gurgel-Giannetti et al. 2001, 2002; Wallgren-Pettersson et al. 2002). A definite diagnosis of *NEB* related disease was most often achieved by a combination of denaturing high‐performance liquid chromatography and Sanger sequencing (Lehtokari et al. 2006). Later, next generation sequencing and microarray analysis became available and resulted in a large improvement in the diagnosis of diseases caused by mutations in *NEB* and other large genes, such as *TTN* (Böhm et al. 2013; Kiiski et al. 2016; Sagath et al. 2018; Scoto et al. 2013; Vasli and Laporte 2013; Zenagui et al. 2018). These techniques were further developed in recent years to improve the capture and coverage of difficult areas, such as repeat regions in *NEB*, and the detection of insertions/deletions as well as copy number variations in *TTN* and *NEB* (Kiiski et al. 2016; Sagath et al. 2018; Zenagui et al. 2018).

Decades of dedicated research and collaborations between geneticists, clinicians and biomedical scientists have resulted in the discovery of a large number of mutations in *NEB* associated with nemaline myopathy and related congenital myopathies (distal myopathy, rod-core myopathy and a myopathy with both caps and nemaline rods; Lehtokari et al. 2011; Piteau et al. 2014; Romero et al. 2009; Wallgren-Pettersson et al. 2007). Most mutations identified to date result in frameshift, premature stop codons or splicing changes causing truncation or deletions within the protein (Pelin et al. 2002). Missense mutations appear to be rare (Lehtokari et al. 2006; Pelin et al. 2002). Mutations are distributed throughout the gene and, to date, no mutation hotspots have been discovered (Donner et al. 2004). *NEB* transcripts are extensively spliced, thus frameshift mutations likely just abolish the expression of some nebulin isoform (also see “Protein structure of nebulin” section; Donner et al. 2004). Thus, the functional defect caused by various *NEB* mutations is hard to predict. For this reason, most likely, no obvious genotype–phenotype correlations have been identified to date (Malfatti et al. 2014). However, severe cases were associated with a large degree of myofibrillar dissociation and markedly reduced contractile performance, while the abundance of rods was inversely correlated with the disease severity (Malfatti et al. 2014).

A recurrent 2502 bp deletion in *NEB*, removing exon 55 and parts of the adjacent introns 54 and 55, exists in individuals from the genetically homogeneous Ashkenazi Jewish population (carrier frequency: 1 in 108) which was later also discovered in other cases around the world (Anderson et al. 2004; Lehtokari et al. 2009). Mutations that cause a loss of function in only one *NEB* allele appear to not lead to disease in humans and mice (*Neb* knock out; KO) suggesting one *NEB*/*Neb* allele is sufficient to produce normal protein levels (Gineste et al. 2013). A comprehensive review of *NEB* mutations was published in Lehtokari et al. (2014).

## Genetic and protein structure of nebulin

### Genetic structure of nebulin

In humans, the *NEB* gene, which is located on chromosome 2 (Stedman et al. 1988; Zeviani et al. 1988), consists of 183 exons spanning 249 kb in the human genome (Donner et al. 2004). The full length human nebulin cDNA sequence of 20.8 kb was first isolated and characterised in 1995 (Labeit and Kolmerer 1995) and was predicted to encode a peptide of 6669 residues and a mass of 773 kDa. Later studies showed that nebulin’s size ranges between 600 to 900 kDa and the isoform described in Labeit and Kolmerer (1995) was suggested to correspond to the shortest nebulin sequence (Labeit and Kolmerer 1995; Wang and Williamson 1980; Wright et al. 1993). This size variation is due to extensive alternative splicing as well as copy number variations in a 32 kp triplicate region within the *NEB* (8 exons are repeated 3 times, exon 82–89, 90–97, 98–105) (Bjorklund et al. 2010; Buck et al. 2010; Donner et al. 2004; Laitila et al. 2012). Alternative splicing depends on the muscle type and the developmental stage. Frequently, multiple isoforms were found to be present at the same time (Donner et al. 2004; Laitila et al. 2012; Lam et al. 2018).

The mouse *Neb* gene was found to contain 165 exons in a 202 kb DNA segment (Kazmierski et al. 2003). Alternative splicing was also detected in mouse muscle and patterns were found to be similar to those described in human muscle (Buck et al. 2010).

### Protein structure of nebulin

**Fig. 2:**
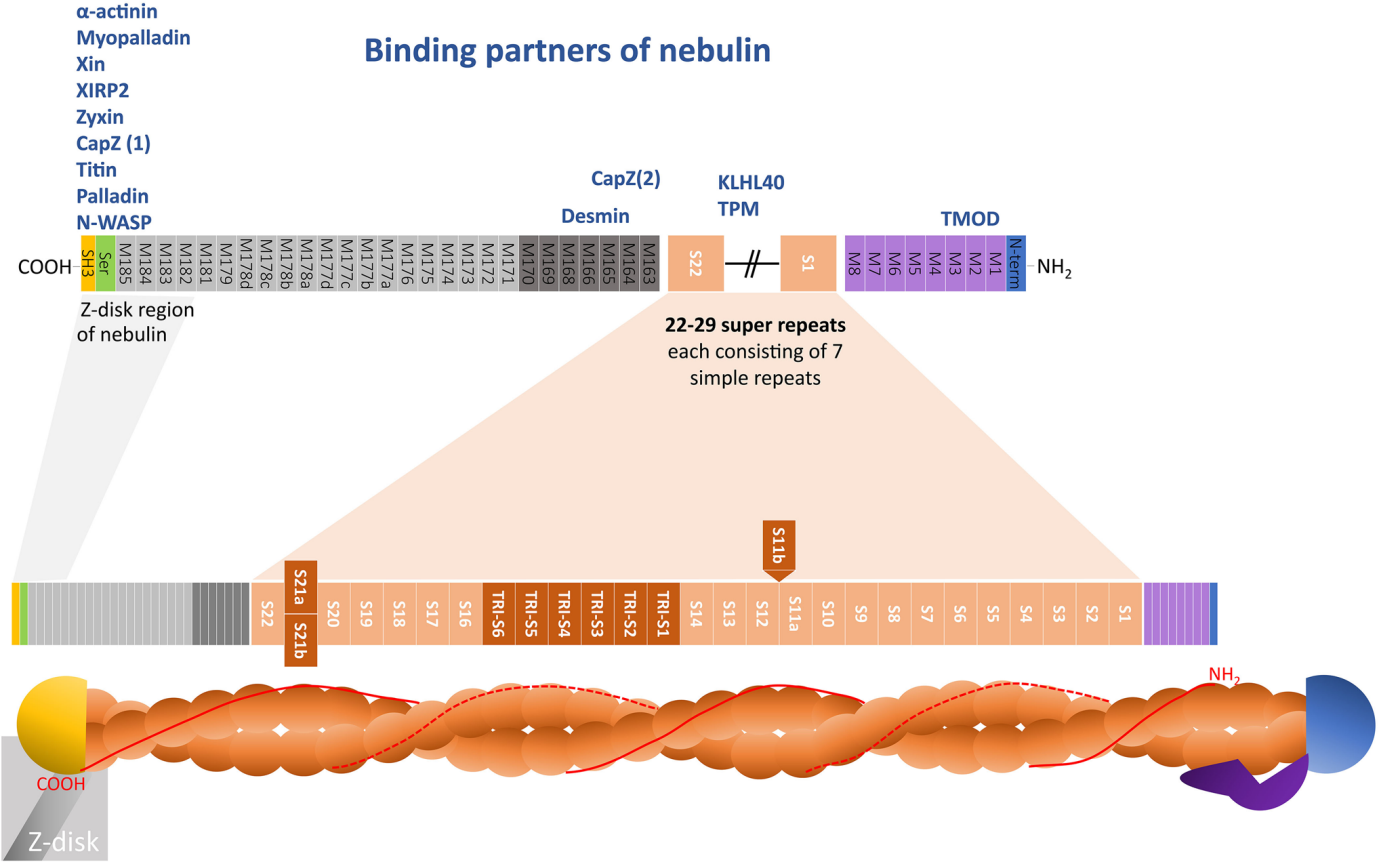
Nebulin protein structure and binding partners. The majority of nebulin’s protein sequence consists of repetitive modules (M) called simple repeats which correspond to actin binding sites. The central region is further organised into super-repeats made up of seven simple repeats each (orange). The composition of the central super-repeat region is strongly affected by alternative splicing, varying the number of super-repeats in the produced protein from 22 to 29. Three main areas have been found to be alternatively spliced (shown in dark orange): (1) exons 63–66 (encoding S11b; transcripts were found to either contain or lack this super-repeat); (2) exons 82–105 [the triplicate region of nebulin encodes 6 super-repeats (TRI-S1 to TRI-S6); the exact splicing pattern has not been established]; (3) exons 143/144 (encoding S21a or S21b; both exons have not been detected in the same transcript). Within the central super-repeat region nebulin is thought to interact with tropomyosin and KLHL40 (listed above in blue). The N- and C-terminus of nebulin are not organised into super-repeats. The C-terminus is made up of two distinct versions of repeats called linker repeats (M163–M170; dark grey) and simple repeats (M171–M183; light grey) (Labeit and Kolmerer 1995). Nebulin’s C-terminus localises within the Z-disk of the sarcomere, likely from M182. A serine-rich region and a highly conserved Src homology-3 (SH3) domain are located at the C-terminus of nebulin and mediate interactions with a large number of proteins (listed above in blue). The N-terminus of nebulin is close to the pointed end of the thin filament. The first 77 N-terminal residues contain a unique, glutamic acid rich sequence (blue) followed by repeat M1–M8 (purple) which are distinct from the remaining repeats and important to mediate tropomodulin interactions at the thin filament pointed end. (Color figure online)

Protein sequence analysis of nebulin showed that the majority of nebulin’s amino acid sequence consists of repeat modules of 35–40 residues containing an SDXXYK motif, which are called simple repeats (Fig. 2; Labeit and Kolmerer 1995). Depending on the splice isoform, the number of simple repeats was suggested to vary from 179 to 239 (Pelin and Wallgren-Pettersson 2008). Each repeat is ~ 5.5 nm apart and was suggested to be largely α-helical in structure (Jin and Wang 1991b; Labeit et al. 1991; Pfuhl et al. 1994). The simple repeats were found to correspond to actin binding sites (thus the largest nebulin molecule can likely associate with 239 actins; Pelin and Wallgren-Pettersson 2008). Repeat motifs, like nebulin’s simple repeats, are also present in a number of other proteins, which are thus grouped together into the nebulin protein family (e.g. N-RAP Luo et al. (1997), Lasp-1 Schreiber et al. (1998), p80/85 Wu et al. (1991), Lasp-2 Li et al. (2004) and nebulette Moncman and Wang (1995)).

#### Central super-repeat region of nebulin

The central part of nebulin is organised into super-repeats made up of seven simple repeats each (Labeit and Kolmerer 1995). Each super-repeat spans 38.5 nm and contains a conserved WLKGIGW motif. Since this structure corresponds to the arrangement of troponin and tropomyosin along the thin filament, it was presumed that the conserved motif constitutes a troponin/tropomyosin interaction site (Jin and Wang 1991a, b; Labeit and Kolmerer 1995; Pfuhl et al. 1994). However, while tropomyosin was found to bind nebulin super-repeats in vitro (Marttila et al. 2014), the exact binding site remains to be established.

In human muscle, the central super-repeat region is strongly affected by alternative splicing, varying the number of super-repeats in the produced protein from 22 to 29 (see Fig. 2) (Donner et al. 2004; Laitila et al. 2012; Lam et al. 2018; Pelin and Wallgren-Pettersson 2008). Three main areas have been found to be alternatively spliced: (1) exons 63–66 (encoding super-repeat 11b, S11b; transcripts were found to either contain or lack this super-repeat); (2) exons 82–105 [encoding 6 super-repeats in the triplicate region of nebulin (TRI-S1 to TRI-S6 in Fig. 2); the exact splicing pattern has not been established]; (3) exons 143/144 (encoding S21a or S21b; both exons have not been detected in the same transcript; exon 144 encodes a protein kinase C phosphorylation site which exon 143 lacks).

#### Nebulin’s N- and C-terminus have unique sequences

The N- and C-terminus of nebulin are not organised into super-repeats but have unique sequences imperative to their individual functions.

At the C-terminus of nebulin, two distinct versions of repeats were described and were termed linker repeats (M163–M170; dark grey in Fig. 2) and simple repeats (M171–M183; light grey in Fig. 2) (Labeit and Kolmerer 1995). Nebulin’s C-terminus localises within the Z-disk of the sarcomere. Exactly how far nebulin penetrates into the Z-disk and whether two nebulin molecules from neighbouring sarcomeres overlap is controversial (reviewed in Ottenheijm et al. 2012). Labeit and Kolmerer (1995) suggested that nebulin inserts into the Z-disk with M171 (the first simple repeat). However, electron microscopy studies suggested that nebulin’s C-terminus is inserted into the Z-disk, starting with M182 (Millevoi et al. 1998). If nebulin’s region M182 through to the C-terminus is located in the Z-disk, theoretical models propose that molecules from either side would overlap by about 75 nm in the sarcomere center (overlap model) or only penetrate 25 nm into the Z-disk leaving the center of the Z-disk nebulin free (no-overlap model), depending on whether the Z-disk region of nebulin is α helical or unstructured (Millevoi et al. 1998). In a third study, M160–M164 were suggested to be located within the Z-disk due to the presence of a CapZ binding site within these modules (Pappas et al. 2008). The authors proposed an alternative Z-disk arrangement of nebulin. In their model, nebulin penetrates the entire Z-disk and cross-links thin filaments from neighbouring sarcomeres (cross-linking model). Which of these models is correct remains to be experimentally tested.

The far C-terminus of nebulin has a serine-rich region followed by a Src homology-3 (SH3) domain. The SH3 domain is highly conserved (Politou et al. 1998) and has been found to mediate the interaction with a number of proteins (see “Binding partners of nebulin” section).

Alternative splicing was also found to affect simple repeats M176–M182 (exons 166–177) and differential splicing in this region was suggested to contribute to differences in Z-disk width in various muscle types (Donner et al. 2004).

The N-terminus of nebulin is close to the pointed end of the thin filament (Wright et al. 1993). The first 77 N-terminal residues contain a unique, glutamic acid rich sequence followed by repeat M1–M8 which are distinct from the remaining repeats (Labeit and Kolmerer 1995) and important to mediate tropomodulin (Tmod) interactions at the thin filament pointed end (also see “Binding partners of nebulin” section; Fowler 1997).

### Nebulin protein expression

The protein product of *NEB* is predominantly expressed in skeletal muscle, but low levels of expression (0.2% of nebulin found in skeletal muscle) were also detected in cardiac muscle (Bang et al. 2006; Donner et al. 2004; Kazmierski et al. 2003). Approximately 50% of atrial cardiomyocytes express nebulin, whereas it is present in only few ventricular cardiomyocytes (Bang et al. 2006). Additional tissues with low abundance expression of nebulin with unknown functional relevance are aorta, liver (Bang et al. 2006) and brain (Laitila et al. 2012).

### Posttranslational modifications

To date, the knowledge concerning posttranslational modifications on nebulin is limited. One study suggested that nebulin protein was extensively and dynamically phosphorylated on serine and threonine residues in mouse diaphragm and that this phosphorylation was increased upon isoproterenol treatment (β-adrenergic stimulation) (Somerville and Wang 1988). Differences in the amount of phosphorylation in mouse tibialis anterior and soleus were also detected in adult versus neonatal muscle (Buck et al. 2010). Not much is known about how these modifications may affect nebulin function. However, two phosphorylation sites within the serine-rich region N-terminally of the SH3 domain within the Z-disk were suggested to regulate the interaction of nebulin with N-WASP in an IGF-1 (PI3K-Akt) dependent manner. In brief, phosphorylation of these sites reduced binding of N-WASP. When N-WASP is bound it was suggested to result in Z-disk localisation and be involved in the formation of actin filaments upon IGF-1 stimulation (Takano et al. 2010).

### Nebulin–thin filament interaction

Nebulin binds polymeric actin and acts as a template protein for the thin filament in the skeletal muscle sarcomere (Jin and Wang 1991a, b). Analysis of the nebulin/MHC ratio in skeletal muscle indicated that there are ~ 2.8 nebulin molecules per thin filament (taking into account the number of MHC molecules and that there are twice as many thin filaments than thick filaments; Buck et al. 2010). Considering the helical symmetry of the thin filament, an even number is most likely. Therefore, it is generally assumed that two nebulin molecules associate with one thin filament (Fig. 1; Buck et al. 2010; Labeit et al. 1991; Wang and Wright 1988). The nebulin binding site on the actin filament has been localised, by chemical crosslinking studies, to the N-terminal region of actin, in proximity of tropomyosin and myosin binding sites (Shih et al. 1997). Modelling data, however, place nebulin in the phalloidin binding groove of actin (Pfuhl et al. 1994) suggesting that nebulin may be able to shift position on the actin filament (Wang et al. 1996). This is further supported by staining experiments, which suggest that nebulin and phalloidin compete for the same binding site on actin filaments (Ao and Lehrer 1995). Electron microscopy studies with nebulin fragments suggest three binding positions for nebulin on actin [two on subunit 1 (one involving the N-terminus of actin) and one on subunit 4] and the possibility that nebulin is able to “roll” or “twist” on the surface of actin (Lukoyanova et al. 2002). Such movement of nebulin on the surface of actin has not yet been established in X-ray diffraction experiments, but nebulin’s backbone may be too slender to detect (Kiss et al. 2018).

A number of studies have been performed to decipher the actin binding properties and structure of nebulin. Since isolating full length nebulin was challenging (due to its enormous size), studying fragments of nebulin containing one or more of the 35 amino acid repeats was the strategy of choice for many studies (Chen et al. 1993; Jin and Wang 1991a, b; Pfuhl et al. 1994, 1996; Root and Wang 1994, 2001). These studies have suggested that not all nebulin modules bind actin molecules with the same affinity. Nebulin was proposed to make one high affinity contact for every seven actin monomers. The remaining contact points display lower affinity (Root and Wang 1994). Additionally, the binding affinity of individual super-repeats was found to vary. Super-repeats 1–4 at the C-terminus and super-repeat S22 at the N-terminus were found to have stronger affinity than the central super-repeats (Laitila et al. 2019). The super-repeat/actin binding affinity was suggested to correlate with the helical propensities of the individual repeat (Pfuhl et al. 1996). It is likely that these differences in affinity are of importance to the function of nebulin. For instance, differences in binding affinity may mediate how nebulin associates with the actin filament. Nebulin was proposed to bind to the thin filament with a zipper-like mechanism, starting from high affinity actin binding modules close to the Z-disk and extending to the pointed end (Chen et al. 1993; Pfuhl et al. 1996). Whether this model is indeed correct and what role the high affinity binding module near the pointed end plays during nebulin association remains to be experimentally established.

Isolation of full length nebulin enabled investigations of its structure and mechanical properties (Chitose et al. 2010; Yadavalli et al. 2009). In the absence of actin, nebulin was suggested to form particles of about 20 nm in diameter (Chitose et al. 2010). Whether this appearance represents a physiological conformation of nebulin remains to be established. Once bound, nebulin fragments were found to be extremely elongated and likely adapted a structure other than an α-helix (e.g. a 3_10_-helix) (Root and Wang 2001). Its elastic properties are likely to exert a compressive force on the actin filament thus stiffening it and protecting it against lateral stress (Yadavalli et al. 2009).

### Binding partners of nebulin

Nebulin interacts with a number of other proteins (see Fig. 2), and thus plays an important role in interconnecting the thin filament with various other proteins and filament systems. In addition to interacting with actin, the central, super-repeat region of the molecule also binds directly to myosin and tropomyosin and altered binding affinity for these proteins has been found when investigating nebulin protein affected by nemaline myopathy causing *NEB* mutations (Marttila et al. 2014). Additionally, KLHL40 and 41 are thought to bind and be involved in regulating protein levels of nebulin at an as yet undefined location of the molecule (Garg et al. 2014; Ramirez-Martinez et al. 2017).

Nebulin’s N-terminus interacts with Tmod (McElhinny et al. 2001). This interaction was suggested to be critical for Tmod localisation to the pointed end, where it acts as a thin filament capping protein regulating dynamic exchange of actin monomers (Weber et al. 1994, 1999). The evidence that nebulin is important for Tmod localisation stems from nemaline myopathy patients with *NEB* mutations (exon 55, resulting in reduced nebulin levels) and *Neb* KO mice. Both show abnormal Tmod localisation (not at the pointed end of the thin filament, but instead along the filament closer to the Z-disk) (Ottenheijm et al. 2009). However, when a small version of nebulin (mini-nebulin) was introduced which contained the N-terminal Tmod binding site, Tmod staining was detected at thin filament pointed ends, but not necessarily in the vicinity of the N-terminal Tmod binding site on nebulin (Pappas et al. 2010). It is possible that the abnormal Tmod localisation observed in patients with *NEB* mutations and *Neb* KO mice is due to thin filaments with variable lengths instead of nebulin loss. It remains to be conclusively established whether nebulin is indeed required for the regulation of Tmod localisation.

Nebulin’s C-terminal SH3 domain interacts with a number of important Z-disk proteins including α-actinin (Chitose et al. 2010; Nave et al. 1990), myopalladin (Bang et al. 2001; Ma and Wang 2002), Xin actin-binding repeat-containing proteins Xin and XIRP2 (proteins associate with myofibril-membrane attachment points; Eulitz et al. 2013) and the focal adhesion molecule zyxin (Li et al. 2004). In the case of myopalladin, nebulin appears critical for localising this protein to the Z-disk, as in the Z-disk of *Neb* KO mice the levels of myopalladin were greatly reduced (Witt et al. 2006).

The C-terminus of nebulin also binds to the barbed end thin filament capping protein CapZ (also called β-actinin) via two distinct sites (see Fig. 2) (Pappas et al. 2008; Witt et al. 2006). It was suggested to anchor CapZ in the Z-disk, as *Neb* KO mouse show altered CapZ distribution (Witt et al. 2006). Repeat 163–170 interact with desmin, a protein of the intermediate filament system. Via this interaction, nebulin links the intermediate filament system to the myofibril, which was suggested to be important for the lateral organisation of adjacent Z-disks (Bang et al. 2002; Tonino et al. 2010). Additional lateral support may be provided via Archvillin, a protein of the costameres, which binds to nebulin’s residues 6457–6528 close to the C-terminus (Lee et al. 2008).

Nebulin also interacts with titin, via two domains: (1) the Zis1 region of titin is able to interact with repeat 185-SH3 of nebulin. Both are localised in close proximity within the Z-disk, so this interaction is likely to occur in vivo (Labeit et al. 2006; Witt et al. 2006); (2) another potential interaction was identified between the PEVK segment of titin and the SH3 from nebulin (Ma and Wang 2002). The biological significance of this interaction is unclear as these two domains do not colocalise in the sarcomere.

## Nebulin has diverse roles in skeletal muscle

Nebulin is a giant protein, which fulfils a number of important functions in skeletal muscle. Decades of intensive research involving the generation of multiple nebulin animal models (Table 1) have succeeded in (at least partially) lifting the “nebula” around this protein. These studies aimed to obtain a detailed understanding of nebulin’s biology, which is critical for the development of treatment approaches for nebulin-based disease. Below, we discuss the various functions of nebulin and describe how loss of these functions results in skeletal muscle weakness.

**Table 1:** Nebulin animal models

Animal model	Description	Phenotype	Background	Publication
*Neb* KO mouse	Deletion of exon 1 (replacement with Cre recombinase, neomycin and frt) No nebulin expression	Mice die within 8–11 days after birth from muscle weakness, normal sarcomere assembly, 25% shorter thin filaments and reduced force	C57/BE and Black Swiss	Bang et al. (2006)
*Neb*^+/−^ Rosa 26 mice	Β-galactosidase expression in tissues expressing nebulin	Viable and indistinguishable from wild-type litter mates	C57/B6 and Black Swiss Rosa26	Bang et al. (2006)
*Neb* KO mouse	Deletion of Cap site, TATA-box and amino-terminus of nebulin resulting in complete absence of nebulin protein	Growth retardation and severe myopathy. ~ 90% of mice die within the first 2 weeks and the remaining animals die in week 3	N/D	Witt et al. (2006)
*neb* mutant Zebrafish	Point mutation in nebulin IVS43+1G>A, a mutation in the canonical splice donor site abolishes splicing of exon 43 and results in skipping of exon 43 (ENSDART00000061293) No protein expression or protein without N-terminus Exon 43 is the equivalent of exon 78 in human *NEB* (nemaline myopathy patients with mutations in this exon were previously reported)	Progressive loss of motor function, fish die within 5–7 days post fertilisation	N/D	Telfer et al. (2012) generated by ENU mutagenesis at the Sanger Institute Zebrafish Mutant Resource
*Neb* ΔExon55 mouse	In frame deletion of exon 55 to model a founder mutation frequently observed in nemaline myopathy patients, results in severely reduced protein levels of nebulin (~ 2% on post-natal day 5)	Growth retardation and death within 1 week after birth	C57/B6J	Ottenheijm et al. (2013)
*Neb* ΔSH3 mouse	Knock in of a stop codon at the 3′ end of the nebulin serine-rich region (residue I7097, exon 166 encoded by the last exon of nebulin). Truncated nebulin was expressed at levels comparable to wild type	No histological or ultrastructural abnormalities, normal isometric stress, but more vulnerable to eccentric contraction induced injury	C57/BL6	Yamamoto et al. (2013)
*Neb* conditional KO (cKO) mouse	The translational start codon of the *Neb* gene was knock out using the flox-Cre system under the control of the muscle creatine kinase promotor. This results in nebulin protein expression reduction to hardly detectable levels in striated muscle within weeks after birth	50% of mice die within 3 months, most remaining mice survive to adulthood. They display lower body weight, contractile defects, nemaline rods, switch to oxidative fiber type	N/D	Li et al. (2015)
*neb* mutant zebrafish strain sa906	Nonsense mutation in neb exon 30 (of 134)	Reduced swim performance, nemaline bodies and actin accumulations, reduced muscle mass and reduced sarcomeric organisation		Sztal et al. (2018)
*neb* mutant zebrafish strain sa906/Lifeact-eGFP	Nonsense mutation in neb exon 30 (of 134); crossed with Tg (Lifeact-eGFP) transgenic line	Reduced swim performance, nemaline bodies and actin accumulations, reduced muscle mass and reduced sarcomeric organisation; actin in all thin filaments labelled		Sztal et al. (2018)
*Neb*Δ163–165	Stop codons after the start of murine exon 163; complete translation of the final actin-binding module M206, lacking serine rich region and SH3 domain. Resulted in a reduction in nebulin levels in EDL, normal protein levels in SOL	Initial high mortality (within the first week) as judged from a skewed Mendelian ratio. 87% of surviving homozygous mice survived to adulthood Reduced body weight which was due to loss of muscle mass rather than reduced growth (tibia length was unchanged)	C57BL/6J	Li et al. (2019)

### Thin filament length regulation

Possibly the first suggested function of nebulin, and probably one of the most controversially discussed ones, is its role in the regulation of thin filament length. Thin filament structure is tightly controlled by a complex interplay of the various thin filament components. In skeletal muscle, thin filaments are regulated to a uniform length of about 1.0–1.3 µm, depending on species and muscle type (Gokhin et al. 2012). Thin filaments are composed of actin polymers which are embedded in the Z-disk with the barbed end while the pointed end is located in the centre of the sarcomere. As mentioned above the thin filament is associated with capping proteins at both ends (CapZ at the barbed end Fowler 1996; Schafer and Cooper 1995 and Tmod at the pointed end Fowler 1997) and nebulin is known to interact with both caps. Despite the fact that thin filament length is highly uniform within the muscle fiber, thin filaments are not static structures. Microinjection experiments demonstrated that actin monomers and other thin filament components are exchanged in vivo and that the ends are not tightly capped all the time (reviewed in Littlefield and Fowler 1998). Additionally, thin filament length is able to change in response to sarcomere lengths to allow for an optimal thin/thick filament overlap during muscle contraction (Kolb et al. 2016).

Nebulin was suggested to be involved in thin filament length regulation, because reduced nebulin protein levels result in abnormal thin filament lengths in *NEB*-related nemaline myopathy patients as well as in nebulin deficient mouse models, zebrafish and chick skeletal myocytes (Bang et al. 2006; de Winter et al. 2016; Ottenheijm et al. 2009, 2010, 2013; Pappas et al. 2008; Telfer et al. 2012; Witt et al. 2006). Uniform thin filament lengths are critical for an optimal overlap between the thin and the thick filaments facilitating the formation of an optimal number of myosin cross-bridges during muscle contraction (Granzier et al. 1991). Dysregulation of thin filament length in nebulin deficient or nebulin mutant muscle was shown to alter the length–tension relationship of muscle, and consequently impair force generation at longer muscle lengths (de Winter et al. 2016; Gokhin et al. 2009; Ottenheijm et al. 2009). In cardiac muscle, nebulin expression is very low and it is controversial whether nebulin contributes to thin filament length regulation in the heart. Nebulin deficiency in vivo did not result in abnormal thin filament length (Kolb et al. 2016).

Despite nebulin being required for normal thin filament lengths, the exact role of nebulin in regulating thin filament length was controversially discussed for more than 20 years (Fowler et al. 2006; Horowits 2006; Lin et al. 2017; Littlefield and Fowler 1998, 2008). Nebulin was at first believed to regulate thin filament length by acting as a molecular ruler—regulating actin filaments to a precise length matching its own. This was supported by multiple lines of evidence: (1) thin filament length in various muscle types correlated with nebulin size (Kruger et al. 1991; Labeit et al. 1991); (2) nebulin was predicted to have a length of about 1 µm and to match the structure of the actin filament containing binding sites for the regulator complex (Kruger et al. 1991); (3) the physiologically low levels of nebulin in cardiac sarcomeres result in more variable thin filament lengths (Wang and Wright 1988); and (4) the N-terminal region of nebulin interacts with the capping protein Tmod (McElhinny et al. 2001) acting as a likely mechanism to halt thin filament extension at nebulin’s end. Alternative models to the ruler hypothesis such as the ‘cap locator’ were also proposed. Hereby, nebulin is involved in length regulation by interacting with Tmod at its N-terminus resulting in a local increase in Tmod concentrations at a specific distance from the Z-disk (Fowler et al. 2006).

However, detailed studies of thin filament lengths did not support that nebulin precisely regulates thin filament length to match its own length. Castillo et al. (2009) showed that thin filament length is typically 0.1–0.3 µm beyond the nebulin N-terminus (Fig. 1) and that nebulin size is unable to account for variable thin filament size in various muscles. In humans, TMOD, which localises to the pointed end of the thin filament, was also found not to colocalise with the nebulin N-terminus, suggesting up to 34% of the thin filament length is nebulin free (depending on the muscle type) (see Fig. 1; Gokhin et al. 2012). Additionally, replacement of full length nebulin (~ 700 kDa) with a nebulin protein of reduced size (mini-nebulin, ~ 250 kDa) did not result in adjustment of the thin filament length to the new size of nebulin (Pappas et al. 2010). However, expression of mini-nebulin did result in thin filaments that were on average shorter and did protect the thin filament from latrunculin A induced depolymerisation to lengths shorter than its own length (Pappas et al. 2010). Thus, a model evolved which suggested a combined mode of thin filament length regulation: nebulin stabilises the thin filament core and specifies a minimum length. A nebulin independent mechanism regulates the dynamics of the pointed end beyond nebulin and sets the final length of the thin filament (Castillo et al. 2009; Littlefield and Fowler 2008). This model is supported by nebulin’s ability to reduce actin turnover, accelerate actin nucleation and reduce depolymerisation, thus increasing the stability of the actin polymer and dictating a minimal length (Chen et al. 1993; Pappas et al. 2010).

The hypothesis that other factors are likely involved in regulating thin filament lengths is supported by the inability of purified nebulin to support the formation of uniformly sized actin filaments in vitro (Chitose et al. 2010). In fact, some mutations in *ACTA1*, encoding skeletal actin, also result in shorter thin filaments (de Winter et al. 2016), suggesting that the properties of the actin polymer itself are also critical for thin filament length determination.

Thus, the current model of thin filament length regulation is a ‘two segment mechanism’. Hereby, the proximal segment from the Z-disk is stabilised by nebulin, while a dynamic distal segment is regulated in length by thin filament capping proteins from the Tmod family including TMOD1, TMOD4, leiomodin (LMOD) proteins and potentially other factors regulating actin dynamics (Gokhin and Fowler 2013).

Interestingly, one study has shown that *Neb* conditional KO (cKO) mice are able to (partially) compensate for altered thin filaments by adding additional sarcomeres in series (de Winter et al. 2016). This mechanism allows nebulin deficient muscle to operate at a shorter working sarcomere length in vivo, resulting in optimal thin–thick filament overlap, despite shorter thin filament lengths. Whether this compensation also happens in humans remains to be established, but it certainly calls into question how much of the force deficit observed in *NEB*-nemaline myopathy patients and *Neb* animal models is in fact due to altered thin filament lengths. The mechanism which leads to the addition of sarcomeres in series remains to be unravelled, but it is possible that it involves a machinery which is able to sense and optimise filament overlap. It is unclear if this mechanism is able to control thin filament length or if it operates via other mechanisms such as sarcomere length. A recent computational analysis study suggests, that nebulin’s structure disorder profile is potentially able to sense and fine tune sarcomere overlap (Wu et al. 2016). However, since the compensation in the mouse model took place in the absence of nebulin, it must have involved other proteins.

### The C-terminus of nebulin is critical for sarcomere organisation, Z-disk structure and lateral alignment of myofibrils

Nebulin was proposed to regulate Z-disk width in skeletal muscle, as supported by abnormal Z-disk widths in nebulin deficient mice (Tonino et al. 2010; Witt et al. 2006) and humans (Ottenheijm et al. 2009). To fulfil this function, it has been proposed that the part of nebulin inserted in the Z-disk is alternatively spliced resulting in a variable number of Z-disk modules (also see above, Donner et al. 2004), which correlated with Z-disk widths in various human, rabbit and mouse muscles (Buck et al. 2010; Millevoi et al. 1998). However, it should be noted that if nebulin inserts into the Z-disk at M182, the alternatively spliced modules M176–M182 are not within the Z-disk. Other proteins such as titin and α-actinin likely collaborate with nebulin to regulate Z-disk structure (reviewed in Littlefield and Fowler 1998). For example, the number of Z-repeats in titin also correlates with Z-disk width, potentially because it determines the number of α-actinin cross-links along the thin filament (reviewed in Littlefield and Fowler 1998).

As discussed above, within the Z-disk nebulin interacts with a large number of structural proteins (including α-actinin, proteins of the intermediate filament system, costamere proteins, etc.) thereby stabilising Z-disk structure and cross linking neighbouring myofibrils (Bang et al. 2002; Conover et al. 2009; Tonino et al. 2010). This is critical for maintaining Z-disk structure and myofibril alignment, in particular during muscle stretch and activation. Nebulin deficient muscle shows an increase in lateral displacement between myofibrils (which was more pronounced at larger sarcomere lengths) and in sarcomere disruption during forceful contractions (Kawai et al. 2018; Tonino et al. 2010). Myofibrillar misalignment and myofibril splitting as well as fragmented Z-disks were often observed in *Neb* KO mice and these abnormalities were only present once muscle had been active after birth (Bang et al. 2006). Since structural organisation is highly important for the contractile function of skeletal muscle, this will result in a force deficit. In gastrocnemius muscle of a *Neb* KO mouse, a functional decline in performance, observed from post-natal day 1 to day 7, was accompanied by structural deterioration, in particular, disruptions of the Z-disk (Gokhin et al. 2009). Similarly, repeated activations resulted in a decline in force production in *Neb* KO mice, but not in wild-type litter mates (Gokhin et al. 2009).

The question of whether these structural functions of nebulin are solely fulfilled by nebulin’s C-terminal, Z-disk associated domains, was recently tackled with the development of two mouse models (also see Table 1): (1) lacking the SH3 domain (ΔSH3; Yamamoto et al. 2013) and (2) a C-terminal truncation after the final actin binding module, removing the serine rich region and the SH3 domain (NebΔ163–165; Li et al. 2019). Interestingly, both, the serine rich region and the SH3 domain are dispensable for the incorporation of nebulin into the Z-disk and the thin filament as well as thin filament length regulation in soleus and EDL muscles (Li et al. 2019). This suggests that the actin binding modules are sufficient to fulfil these functions. The mouse lacking only the SH3 domain also showed normal muscle myogenesis, regeneration, contractility and no evidence of structural abnormalities such as Z-disk dissolution, streaming or misalignment (Yamamoto et al. 2013). The mouse lacking both, the serine-rich region and the SH3 domain, did however show dysregulated Z-disk widths and Z-disk protein aggregates (Li et al. 2019). It has to be noted, that the NebΔ163–165 resulted in reduced nebulin levels in EDL muscle which showed more sarcomeric dysorganisation, whereas normal nebulin expression was observed in NebΔ163–165 soleus and the NebΔSH3 mouse model (Li et al. 2019; Yamamoto et al. 2013). Thus it cannot be excluded that the structural defects relate, at least in part, to reduced nebulin protein levels rather than the absence of the serine-rich region and the SH3 domain. Both mouse models only show mild muscle dysfunction at baseline. In the *Neb*Δ163–165 mouse model (lacking the C-terminal domains) a deficit in contractility in intact soleus muscles with preserved nebulin protein levels suggested that the C-terminus is important for normal force generation (Li et al. 2019), while just deleting the SH3 domain did not result in abnormal contractile force (Yamamoto et al. 2013). Conditions of increased strain on the muscle (eccentric contractions) resulted in more muscle damage and force loss in both mouse models than in wild-type littermates suggesting that deleting as little as the SH3 domain results in reduced ability of the muscle to cope with contractile strain (Li et al. 2019; Yamamoto et al. 2013).

### Nebulin during actin filament formation and maintenance

The development of mature striated muscle sarcomeres requires the highly coordinated assembly of a large number of proteins into repeating patterns. Nebulin shows a striated pattern during myofibrillogenesis after the formation of I–Z–I bodies (containing actin and α-actinin) (Komiyama et al. 1992) but before thin filaments achieve their mature lengths (Moncman and Wang 1996; Nwe et al. 1999; Shimada et al. 1996). Targeting of nebulin to I–Z–I bodies in immature sarcomeres in the correct orientation was proposed to require interactions with the PEVK domain of titin and myopalladin which likely help to tether nebulin’s C-terminus to the Z-disk (Ma and Wang 2002). Additionally, single molecule nano-mechanics measurements suggest that nebulin requires mechanical loading of at least 300 pN to achieve an elongated state which can be incorporated into the thin filament (Yadavalli et al. 2009). This mechanical load was hypothesised to originate from myosin heads (one myosin head can generate about 2–5 pN, so it would take about 100—note one half thick filament has ~ 300), thus the authors suggest nebulin is incorporated after formation of A-bands and I–Z–I bodies (Yadavalli et al. 2009).

Whether nebulin is critical for the process of sarcomere formation is somewhat controversial. On one hand, primary skeletal myoblasts in a nebulin knock-down cell culture model were unable to develop into striated myotubes (McElhinny et al. 2005). However, on the other hand, both *Neb* KO mice and *neb* mutant zebrafish are able to form sarcomeres with largely normal structure (Bang et al. 2006; Sztal et al. 2018). Ottenheijm et al. (2009) also showed that the number of thin filaments was not reduced in nebulin-related nemaline myopathy patients (deletion exon 55, causing reduced nebulin levels) suggesting that thin filaments were formed with normal efficiency despite the presence of reduced nebulin levels. Thus, in vivo evidence suggests that a nebulin independent mechanism of sarcomere formation exists. In *Neb* KO mice, the length of thin filaments is also uniform but 25% shorter at birth. Thus, thin filament lengths are also regulated in the absence of nebulin (Bang et al. 2006).

Progressive shortening and the appearance of thin filaments of non-uniform length, as well as progressive disruption of myofibrillar and Z-disk structure, only appear after birth. This indicates that nebulin plays an important role in regulation and maintenance of the structure of the contractile apparatus (Bang et al. 2006). At 10 days of age *Neb* KO mice show shorter thin filaments ranging in size from 0.4 to 1.2 µm (Witt et al. 2006). Similarly, despite the muscle forming initially, the *neb* mutant zebrafish displays reduced muscle fiber growth (Sztal et al. 2018).

Thus, although nebulin is most likely not critical for initial sarcomere formation, it has been suggested that it does target N-WASP to the Z-disk and the two proteins facilitate actin filament formation as a hypertrophy response (Takano et al. 2010). However, two independent studies were not able to reproduce this result (Li et al. 2019; Yamamoto et al. 2013). The reason for this discrepancy is currently poorly understood and warrants further investigations.

### Cross-bridge cycling

During skeletal muscle contraction, a cyclical interaction between thick filament myosin heads and the thin filament (cross-bridge cycling) results in sliding of the filaments past each other and thus muscle shortening (Huxley 1957). This process is powered through the hydrolysis of adenosine triphosphate (ATP) to adenosine diphosphate (ADP) and *ortho*phosphate (P_i_) (Huxley 2000). The hydrolysis is catalysed by the myosin heavy chain ATPase on the myosin head and constitutes the rate limiting step of actin–myosin cross-bridge cycling.

#### Nebulin deficiency results in skeletal muscle contractile dysfunction

Nebulin deficient patients and various animal models with nebulin deficiency show substantial contractile dysfunction. The results of studies assessing sarcomeric function in nebulin mutant or nebulin deficient muscle are summarised in Table 2. The force deficit measured varied widely between studies. In *Neb* KO, cKO and *Neb*Δ55 models contractile forces ranging from 8 to 73% of wild-type values were measured, depending on the age, muscle type and methodology of the study. Human *NEB*-nemaline myopathy patients with various *NEB* mutations produced 7–69% of the force measured in control patients.

**Table 2:** Summary of contractile mechanics studies on nebulin deficient or nebulin mutant muscle

Model/muscle type/age	Experimental setup	SL (μm)	Finding						Publication
			Specific force deficit (% of WT)	k_TR_	Tension cost	Force/Ca^2+^ curve	Active stiffness	Other	
*Neb* KO mice/TA/P1	Intact muscle mechanics	N/A	50%	N/D	N/D	N/D	N/D	Reduced thin filament length, excitation/contraction coupling was not significantly altered, at this age the contractile machinery displayed normal morphology	Bang et al. (2006)
*Neb* KO mice/TA/P10	Permeabilized fiber bundle contractility	2	N/D?	N/D	N/D	pCa_50_ – *n*_*H*_ ↓	N/D		Witt et al. (2006)
*Neb* KO mice/gastrocnemius/P1 and P7	Intact muscle mechanics	N/A	P1 = 73% P7 = 8%	N/D	N/D	N/D	N/D	Force–SL relationship was shifted to the left, greater force decline during repeated isometric tetani	Gokhin et al. (2009)
*Neb* KO mice/Psoas/P1	Permeabilized fiber bundle contractility thin filament length and structure were comparable to control	2.6	35%	↓	N/D	pCa_50_ – *n*_*H*_ –	↓	Tension/stiffness was normal, conclusion: reduction in the number of motors without affecting the force per motor due to reduced attachment rate	Bang et al. (2009)
*Neb* KO mice/TA/P7-9	Permeabilized fiber bundle contractility; at SL of 2.0 µm both, WT and KO fibers, are predicted to produce 80% of WT Fmax	2.0	~ 50%	↓	↑	pCa_50_ ↓ *n*_*H*_ ↓	↓	Tension/stiffness was normal, no changes in myosin heavy chain isoforms, myosin light chain composition/phosphorylation and troponin and tropomyosin isoforms conclusion: reduction in the number of motors without affecting the force per motor, nebulin has a direct role in modulating calcium sensitivity of the thin filament	Chandra et al. (2009)
4 *NEB*-NM patients	Permeabilised fiber bundles contractility	2.5 (Control), just above slack in *NEB* myofibers	7%	↓	↑	pCa_50_ ↓ *n*_*H*_ –	N/D	Reduced thin filament length	Ottenheijm et al. (2010)
1 *NEB*-NM patient	Permeabilised fiber bundles contractility	2.5 (Control), just above slack in *NEB* myofibers	5%	↓	↑	N/D	N/D		Lawlor et al. (2011)
1 *NEB*-NM patient	Permeabilized fibre contractility	2.70–2.80	69%	↓	N/D	pCa_50_ – *n*_*H*_ –	↓	Normal force–sarcomere length relationship, abnormal myosin light chain composition, tension/stiffness was normal	Ochala et al. (2011)
								Conclusion: reduction in the fraction of myosin heads strongly bound to actin rather than a decrease in the force per myosin cross-bridge	
*Neb*Δ55 mice/TA/P4-7	Permeabilized fibre contractility and individual myofibrils, sarcomere length?	2.2	38% (At SL of 2.4 µm)	↓	↑	pCa_50_ ↓ *n*_*H*_ –	N/D	Force–SL relationship was shifted to the left	Ottenheijm et al. (2013)
4 *NEB*-NM patients	Permeabilised fiber bundle contractility	2.1 and 2.6	17%	N/D	N/D	SL 2.1 pCa_50_ ↓ *n*_*H*_ ↓ SL 2.6 pCa_50_ – *n*_*H*_ N/D	N/D		de Winter et al. (2013)
*Neb*ΔSH3 mouse, EDL (2m, 6m)	Intact muscle mechanics	N/A	100%	N/D	N/D	N/D	N/D	More force loss due to eccentric contractions, less sensitive to electrical stimulation (force/frequency)	Yamamoto et al. (2013)
*Neb* cKO mice soleus, EDL (5w and 6m)	Exercise testing, intact muscle mechanics permeabilized fiber/fiber bundles contractility	2.4	SOL_intact_ (5w) 58%	↓	↑	N/D	↓	Tension/stiffness was normal, reduced exercise performance, reduced thin filament length, reduced fraction of strongly attached cross-bridges	Li et al. (2015)
			SOL_intact_ (6m) 43%						
			EDL_intact_ (5w) 15%						
			EDL_intact_ (6m) 23%						
			SOL_perm_?						
			EDL_perm_?						
*Neb*Δ55 mice (6d)/diaphragm, soleus, EDL, gastrocnemius	Permeabilized fibre and individual myofibril contractility	2.3	40–24%	↓	N/D	pCa_50_ ↓ *n*_*H*_ –	↓	Tension/stiffness was normal, Conclusion: reduction in the number of motors without affecting the force per motor	Joureau et al. (2017)
*Neb* cKO/soleus	Permeabilised fiber bundle contractility	2.5	52%	N/D	N/D	N/D	↓	Ratio active stiffness/rigor stiffness was comparable to wild type	Kawai et al. (2018)
								Conclusion: equal amount of cross-bridges are activated during contraction	
*Neb*Δ163–165	Intact muscle mechanics	N/A	A slight force deficit was observed in soleus, while a drastic loss of force was present in EDL	N/D	N/D	N/D	N/D	More force loss due to eccentric contractions, no change in sensitivity to electrical stimulation (force/frequency)	Li et al. (2019)

*TA* Tibialis Anterior, *KO* knock out, *WT* wild type, *NM* nemaline myopathy, *N*/*D* no data, *N*/*A* not applicable, *EDL* extensor digitorum longus, *SL* sarcomere length; ↓, ↑, – down-regulation, up-regulation, unchanged (compared to wild-type), respectively, *Fmax* tension at saturating Ca^2+^

#### Contractile dysfunction cannot solely be explained by abnormal sarcomere structure

As discussed above, changes in thin filament length and structural disorganisation are an obvious cause of weakness as they will reduce the number of available ordered myosin/actin interactions. However, by limiting the influence of abnormal thin filament lengths and sarcomeric disorganisation on contractility measurements, a number of studies provided strong evidence that nebulin is also directly involved in regulating actin-myosin cross-bridge cycling (see Table 2; Bang et al. 2006, 2009; Chandra et al. 2009; Joureau et al. 2017; Kawai et al. 2018; Lawlor et al. 2011; Lee et al. 2013; Li et al. 2015; Ochala et al. 2011; Ottenheijm et al. 2010, 2013). For example, contractile dysfunction persisted at shorter sarcomere lengths at which the filament overlap allows for efficient force generation (Chandra et al. 2009; Ottenheijm et al. 2010), and in models with thin filament lengths and sarcomeric structure comparable to healthy muscle (Bang et al. 2009; Ochala et al. 2011). Kawai et al. (2018) determined that a force deficit of 69% remains after correcting for the lack in overlap in nebulin deficient fibers (which caused a theoretical reduction in force generating cross-bridges of ~ 75.4% in *Neb* KO compared to wild-type). Contractile measurements on structurally intact individual myofibrils clearly showed that structural damage of nebulin deficient muscle was not the only factor in causing muscle weakness, but that contractile dysfunction is intrinsic to the contractile apparatus (Ottenheijm et al. 2013). A recent study using computational modelling provided additional evidence that shorter and nonuniformly distributed thin filament lengths are insufficient to explain the contractile abnormalities observed in nebulin deficient fibers (Mijailovich et al. 2019).

#### Nebulin deficiency results in altered cross-bridge cycling kinetics

Using permeabilised muscle preparations, which allows for direct activation with Ca^2+^ in the bathing solutions, the sarcomeric function and various cross-bridge cycling parameters can be assessed in isolation from upstream excitation contraction (EC) coupling (reviewed in de Winter and Ottenheijm 2017). In nebulin deficient/mutant muscle a decrease in the rate of force re-development after a short period of unloaded shortening (*k*_TR_) was often observed in combination with an increase in the ATP consumption during contraction (tension cost) and a decrease in active fiber stiffness (see Table 2). Assessing the observed contractile parameters in terms of the 2-state kinetic model developed by Huxley (1957) one can determine the contribution of changes in cross-bridge cycling kinetics to the contractile dysfunction. Within this model each myosin motor produces a specific amount of force and thus the total force output of each myofibril depends on the fraction of myosin heads strongly attached to the thin filament. The fraction of attached motor units can be described by the ratio

$${\text{f}}_{\text{app}} /\left( {{\text{f}}_{\text{app}} + {\text{g}}_{\text{app}} } \right),$$

where f_app_ and g_app_ are the rate constant of myosin motor attachment and detachment, respectively. *k*_TR_ is proportional to f_app_ + g_app_. Since ATP hydrolysis (tension cost) is required for myosin head detachment, it is proportional to g_app_ (Brenner and Eisenberg 1986). Taking this into account, the increase in tension cost suggests an increase in g_app_. Since the *k*_TR_ is proportional to f_app_ + g_app_ and g_app_ is increased, f_app_ must be decreased. In summary, nebulin deficient or mutant muscle likely displays a reduced fraction of strongly bound myosin cross-bridges due to a decreased myosin attachment rate and an increase detachment rate (Chandra et al. 2009; Li et al. 2015; Ochala et al. 2011; Ottenheijm et al. 2010). The decrease in active stiffness was proportional to the decrease in force resulting in a maintained tension/stiffness ratio. This suggested that the force per cross-bridge is maintained, but the number of cross-bridges are reduced (Bang et al. 2009; Chandra et al. 2009; Joureau et al. 2017; Li et al. 2015; Ochala et al. 2011). Additional evidence supporting a reduced number of cross-bridges due to nebulin deficiency was obtained using X-ray diffraction, which showed less myosin head recruitment during tetanic contraction in a *Neb* cKO model compared to wild-type muscle (Kiss et al. 2018). Another study found that in the muscle of a patient with *NEB*-related nemaline myopathy the cycling rate of myosin heads attaching to actin is dramatically perturbed, causing a reduction in the fraction of myosin–actin interactions in the strong binding state. This prevented complete thin filament activation, more specifically proper and full tropomyosin movement, further limiting additional binding of myosin cross-bridges (Ochala et al. 2011).

It should be noted that a recent study compared rigor stiffness to active stiffness in *Neb* cKO and wild-type animals to provide an estimation of the number of cross-bridges that are attached during maximal activation compared to the rigor state. They found that the ratio of myofiber stiffness during maximum activation and rigor is similar in *Neb* cKO and wild-type animals (62% vs. 68%, respectively) suggesting that an equal number of cross-bridges are activated during contraction. Thus, Kawai et al. provides evidence which opposes previous studies, suggesting a reduction in the force generated by each cross-bridge instead of a reduced number of cross-bridges as the underlying cause for contractile dysfunction in nebulin deficient muscle (Kawai et al. 2018). However, the data presented by Kawai et al. (2018) was obtained exclusively from type 1 fibers obtained from skinned soleus and the data was not normalised to the total number of myofilaments per muscle cross-section. It remains to be tested whether these factors may partially explain the discrepancy observed between Kawai et al. (2018) and previous work.

Thus, in summary, a growing body of experimental evidence indicates that nebulin is involved in regulating cross-bridge cycling kinetics, and that in the absence of nebulin impaired kinetics contribute to contractile dysfunction.

#### The effect of nebulin deficiency on the force–Ca^2+^ relationship

Many studies have determined the Ca^2+^ sensitivity of contractile force by measuring the force response at incremental Ca^2+^ concentrations to assess the force–pCa relation of myofibers from human or mouse nebulin deficient/mutant muscles. A reduction in the Ca^2+^ sensitivity of force (as described by the Ca^2+^ concentration at which the preparation produces 50% of its maximum force [pCa_50_]) and the cooperativity of Ca^2+^ induced activation (as described by the Hill constant [*n*_*H*_]) was often observed (see Table 2). One study was able to exclude that this effect was caused by differences in regulatory protein isoforms, which have a major influence on the Ca^2+^ sensitivity (Chandra et al. 2009). Some studies found no change in pCa_50_ (Witt et al. 2006), in *n*_*H*_ (Joureau et al. 2017; Ottenheijm et al. 2010, 2013) or in either parameters (Bang et al. 2009; Ochala et al. 2011), but it is unclear whether this was a result of accompanying changes in regulatory isoforms in nebulin-deficient muscle.

Interestingly, a reduced pCa_50_ in nebulin deficient/mutant myofibers compared to control myofibers was found in studies which performed measurements at sarcomere lengths < 2.3 µm (Chandra et al. 2009; de Winter et al. 2013; Joureau et al. 2017; Ottenheijm et al. 2010, 2013), while studies where sarcomere length was set to > 2.4 µm found no change in the pCa_50_ (Bang et al. 2009; de Winter et al. 2013; Ochala et al. 2011) (also see Table 2). De Winter et al. (2013) first noted this relationship between sarcomere length and Ca^2+^ sensitivity in *NEB*-nemaline myopathy patients, as they performed measurements at sarcomere lengths of 2.1 µm and 2.6 µm, and found that Ca^2+^ sensitivity was only perturbed at shorter sarcomere lengths. The authors hypothesised that nebulin deficient myofibers display exaggerated length dependency of activation reminiscent of the physiological properties of cardiac muscle which does not express significant amounts of nebulin (see “Nebulin protein expression” section; de Winter et al. 2013).

#### Mechanisms by which nebulin affects the contractile mechanics of skeletal muscle

The mechanism by which nebulin affects the contractility of skeletal muscle is currently not completely understood. Nebulin fragments containing 7–8 modules from the myosin/actin overlap region (but not modules from the Z-disk region) were found to bind both actin and myosin via distinct binding sites (Jin and Wang 1991a, b; Kruger et al. 1991; Root and Wang 1994). It was proposed that through these interactions nebulin may be important to maintain alignment and lattice spacing of thin and thick filament for optimal interaction. This was supported by a decreased distance between the myosin S1 subunit and actin when nebulin fragment NA3 was present (Root and Wang 1994, 2001). However, a recent X-ray diffraction study did not find altered myosin head position at rest, but less myosin head recruitment during tetanic contraction in a *Neb* cKO model compared to wild-type (Kiss et al. 2018). The process of myosin head recruitment in skeletal muscle relies on the Ca^2+^ induced change in tropomyosin position on the thin filament which partially exposes the myosin binding sites on the actin filament. Initial, weak myosin binding then induces further movement of tropomyosin to allow the formation of strong myosin/actin cross-bridges required for filament sliding (Holmes 1995; McKillop and Geeves 1993; Vibert et al. 1997). In an in vitro motility assay, nebulin was found to be able to inhibit acto-S1 ATPase activity and sliding of filaments in a calmodulin/Ca^2+^ dependent manner. Thus, nebulin by itself might display regulatory functions on thin/thick filaments, similar to tropomyosin (Root and Wang 1994). It is possible that nebulin is able to allosterically enhance binding between actin and myosin by exposing ionic and hydrophobic amino acid residues important for myosin binding (Kawai et al. 2018), resulting in more efficient cross-bridge cycling by increasing the speed of actin–myosin association and decreases cross-bridge detachment. However, nebulin may also act in concert with tropomyosin during thin filament activation. X-ray diffraction data obtained from the Neb cKO model showed altered tropomyosin movement and troponin distribution in the absence of nebulin (Kiss et al. 2018). Whether these changes are a direct effect of nebulin-deficiency or rather an indirect effect of changes in the thin filament helix in nebulin-deficient muscle remains to be investigated. However, computational modelling indicates that changed troponin I detachment from actin could be the underlying mechanism for altered calcium sensitivity of force at long sarcomere lengths (Mijailovich et al. 2019).

Finally, nebulin has been shown to increase thin filament stiffness. X-ray diffraction analysis showed that the actin filament is “stretched” during muscle activation in a tension-dependent manner, as measured by the change in spacing of the 27 Å meridional reflection (representing the actin subunit repeat). The extensibility during contraction is increased in the *Neb* cKO mouse, suggesting that nebulin acts to stiffen the actin filament during contraction (Kiss et al. 2018). Additional evidence for increased thin filament compliance in *Neb* cKO mice were provided by mechanical measurements performed by Kawai et al. (2018). In silico simulations of the effect of reduced thin filament stiffness on cross-bridge cycling suggested that it would lead to reduced speed of force development, with only a small effect on maximal tetanic force (Kiss et al. 2018).

### Nebulin in Ca^2+^ homeostasis and excitation contraction (EC) coupling

In addition to nebulin’s structural and cross-bridge cycling roles, evidence has been presented that it plays a role during Ca^2+^ homeostasis and EC coupling of skeletal muscle. Mechanical testing in intact muscle preparations from the *Neb* ΔSH3 mouse model highlighted a slightly blunted sensitivity to electrical stimulation suggesting the SH3 domain of nebulin may play a role in EC coupling (Yamamoto et al. 2013). Nebulin free fibers also showed a reduced speed of relaxation likely due to a reduced speed of Ca^2+^ uptake (as measured using microsomal homogenates and intact fibers from the flexor digitorum brevis muscle) (Ottenheijm et al. 2008). However, a comparison of the maximum force produced in intact and skinned muscles showed comparable forces. This indicates that abnormal Ca^2+^ handling does not majorly affect the maximal force generating capacity of nebulin deficient muscle (Ottenheijm et al. 2008).

How nebulin affects Ca^2+^ homeostasis and EC coupling is poorly understood. Changes in Ca^2+^ homeostasis and EC coupling may relate to upregulation of sarcolipin [an inhibitor of the sarcoplasmic reticulum (SR) Ca^2+^–ATPase (SERCAs)] in a *Neb* KO mice as shown by gene expression analysis (Gokhin et al. 2009; Witt et al. 2006). Sarcolipin upregulation was found to increase with age, synchronously with the development of ultrastructural abnormalities and reduced contractile performance. This upregulation was also present at the protein level at postnatal day 5 and 15, while other SR-associated proteins (such as phospholamban, calsequestrin) were unchanged and SERCA levels were only slightly reduced (Ottenheijm et al. 2008). How nebulin affects sarcolipin at the transcript and protein level is unclear. An interaction between nebulin and sarcolipin could not be detected (Ottenheijm et al. 2008).

## Treatment

In humans, mutation in *NEB* result in a congenital myopathy, most often nemaline myopathy, associated with significant morbidity and mortality (described in “Nebulin in muscle disease” section). To date, no effective treatment is available for these conditions, but a small number of therapeutic interventions have been investigated. These are reviewed in this section.

### Troponin activators

A number of studies have highlighted that mutations in nebulin lead to a blunted response of the sarcomere to Ca^2+^ induced activation (Chandra et al. 2009; de Winter et al. 2013; Joureau et al. 2017; Ottenheijm et al. 2010, 2013). Thus, the use of troponin activating drugs which increase the force response to Ca^2+^ was investigated.

#### Levosimendan

Levosimendan is a drug which binds and activates slow skeletal/cardiac troponin C (Edes et al. 1995) and is approved for the clinical use in heart conditions (reviewed in Nieminen et al. 2013). It was found to effectively increase the Ca^2+^ sensitivity of contractile force in slow-twitch diaphragm muscle fibers in humans and animal models of heart failure (Doorduin et al. 2012; van Hees et al. 2009, 2011). However, when tested in permeabilised myofibers of patients with *NEB*-related nemaline myopathy, it had no effect on the Ca^2+^ sensitivity of force (de Winter et al. 2015). It is currently unclear what causes the discrepancy between studies, but the authors excluded defective levosimendan or difference in muscle type as a reason (de Winter et al. 2013).

#### CK-2066260

CK-2066260 is a structural analogue of tirasemtiv (formerly CK-2017357), a drug which was in phase 3 clinical trials for amyotrophic lateral sclerosis (VITALITY-ALS). The trial was discontinued due to the drug not meeting the primary or secondary endpoints (https://cytokinetics.com/tirasemtiv/). Like tirasemtiv, CK-2066260 is a fast skeletal troponin activator (Russell et al. 2012). In skinned skeletal muscle fibers of *Neb*Δ55 mice and *NEB* myopathy patients this drug was able to effectively increase the Ca^2+^ sensitivity of force (as measured by the pCa_50_) resulting in an increased tension at submaximal Ca^2+^ which exceeded the tension measured in fibers of wild-type mice (de Winter et al. 2013; Ottenheijm et al. 2013). CK-2066260 was also found to be effective in skinned fast skeletal muscle fibers of *Neb* KO mice, resulting in increased Ca^2+^ sensitivity of force, cross-bridge cycling and maximum tension (Lee et al. 2013). A recent study demonstrated that in addition to enhancing muscle performance, without affecting coordination, CK-2066260 was able to reduce the energetic cost of muscle contraction (Cheng et al. 2019). Thus, this compound presents with many promising attributes and may be useful in treating *NEB*-related skeletal muscle weakness and other related conditions.

### Dietary supplements

Limited, often anecdotal, evidence has suggested the usefulness of a number of dietary supplements for patients with nemaline myopathy. In particular, l-tyrosine has been suggested to have beneficial effects in small-scale trial in humans and in a study using an ACTA1 nemaline myopathy animal model (Nguyen et al. 2011; Ryan et al. 2008). For *NEB* related nemaline myopathy, a study employing a zebrafish model for *neb* nemaline myopathy did not find that the administration of l-tyrosine (10 µM), l-carnitine (10 µM), creatine (100 µM) and taurine (1 mM) had beneficial effect on swim performance and muscle morphology (Sztal et al. 2018). A more detailed study may help improve our understanding of potential benefits dietary supplements may have on humans with *NEB* based nemaline myopathy.

### Myostatin inhibitor ActRIIB-mFc

Recent studies have suggested that myostatin inhibitors are able to improve muscle function in two *Acta1* mouse models (Acta1 p.H40Y and p.D286G; Tinklenberg et al. 2016, 2018). Myostatin is a cytokine released by myocytes and inhibits myogenesis via interactions with the activin type IIB receptors which activates the TGF-β pathway and results in inhibition of cell cycle progression and myofiber hypertrophy. ActRIIB-mFc inhibits the function of myostatin and may thus help ameliorate the myofiber atrophy commonly observed in nemaline myopathy. However, treatment with ActRIIB-mFc from an age of 14 days did result in increased muscle weights in the wild-type mice but not in the *Neb* cKO mice (comparison of treated vs. untreated of each genotype) (Tinklenberg et al. 2019). The study also did not find a significant effect on grip strength, open field, survival or skeletal muscle histology due to myostatin inhibitor treatment on *Neb* cKO mice (Tinklenberg et al. 2019). The authors hypothesised that the lack of efficacy observed did not relate to myostatin signalling, since no evidence of abnormal hypertrophic signalling was found. However, the authors suggest that the severe muscle dysfunction of *Neb* cKO mice may not respond to treatment anymore or that the mechanism of muscle atrophy in *ACTA1* versus *NEB* nemaline myopathy is fundamentally different.

## Discussion and conclusion

In the last ~ 4 decades since nebulin’s discovery this protein has been intensively studied. Despite the challenge of its enormous size, knowledge on nebulin’s function has increased by leaps and bounds, especially due to the development of a number of animal models (Table 1). We now understand that nebulin’s functionality is as enormous as its size and nebulin is involved in a number of critical processes in skeletal muscle, as reviewed in this paper including amongst other things the regulation of thin filament length, structure and mechanical properties as well as actin–myosin cross-bridge cycling during skeletal muscle contraction.

The exact mechanism of how nebulin fulfils some of these functions remains to be elucidated and a number of controversial topics and important unanswered questions remain. For example, is nebulin indeed involved in actin filament formation during muscle hypertrophy? Additionally, many aspects of nebulin’s biology remain unstudied. It is, for instance, currently unknown how rapidly nebulin is turned over in the sarcomere. A recent study on another sarcomeric giant, titin, has provided evidence that this protein is more mobile than would be anticipated based on its enormous size (da Silva Lopes et al. 2011). It remains to be established whether the same is true for nebulin.

The significance of increasing our understanding on the exact function and biology of nebulin is now more readily understandable than ever as next generation sequencing techniques are improving the rate by which disease causing mutations in nebulin are discovered. Currently, no therapy is available for patients suffering from *NEB*-related muscle weakness although some promising therapeutic approaches, such as troponin activating drugs, exist. Additionally, a few treatment strategies have been investigated in other forms of muscle disease which may also be applied to *NEB*-based disease. For example, in a mouse model for *ACTA1*-related nemaline myopathy (His40Tyr) intramuscular injections with an AAV vector containing an alternative myosin light chain (MyLC1_a/emb_) was able to decrease muscle atrophy and thus enhance the contractile performance (Lindqvist et al. 2016). Since abnormal myosin–actin interactions were also found in *NEB*-related muscle weakness this gene therapy approach may be promising. Also, recent advances in the exon skipping field (as reviewed in Echevarria et al. 2018) invite to ponder how this strategy may be applied to *NEB*-related disease. The existence of splice isoforms with various numbers of actin binding super-repeats suggests that skipping of whole super-repeats is well tolerated. It may thus be possible to develop an anti-sense strategy to skip disease causing mutation containing regions in nebulin by removing whole super-repeats. Whether removal of super-repeats is possible remains to be established. In addition, delivery efficacy of gene therapy vectors is still problematic suggesting that any form of gene therapy for *NEB*-related disease is many years away from patient use.

Nonetheless, a detailed understanding of nebulin’s role in skeletal muscle is the most important first step in developing much needed treatments for *NEB*-related myopathies.
